# Mental health policy implementation in low- and middle-income countries: a realist review protocol

**DOI:** 10.1371/journal.pone.0320420

**Published:** 2025-03-25

**Authors:** Rangarirai Matima, Claire van der Westhuizen, Crick Lund, Ferdinand C. Mukumbang

**Affiliations:** 1 Alan J Flisher Centre for Public Mental Health. Department of Psychiatry and Mental Health, University of Cape Town; 2 Centre for Global Mental Health, Health Services and Population Research Department, Institute of Psychiatry, Psychology and Neuroscience, King’s College London; 3 Department of Global Health, School of Public Health, University of Washington, Seattle, Washington, United States of America; University of Siena: Universita degli Studi di Siena, ITALY

## Abstract

**Introduction:**

Formulating and implementing mental health policy is foundational to public mental health. The implementation of mental health policy varies in low- and middle-income countries (LMICs), with some countries having better implementation outcomes than others. Low implementation poses challenges relating to addressing the high burden and wide treatment gaps of mental health conditions. While different implementation strategies are applied to implement mental health policy in different contexts, there is little knowledge of what category of strategies are being used and how and why their implementation produces varied outcomes. To this end, we propose to conduct a realist synthesis to explain how, why, for whom, and under what health system conditions certain policy implementation strategies work or not in LMICs.

**Methods and analysis:**

This paper will detail the protocol on conducting a realist review of the literature on mental health policy implementation in LMICs. Realist reviews/syntheses are theory-driven reviews designed to formulate and test (confirm, refute, or refine) initial programme theories to explain how, why, for whom and under what contexts a programme, intervention or policy works as intended or not. Theory is built by exploring and abstracting context-mechanism-outcome (CMO) configurations in the data. These CMO configurations are identified through retroductive theorizing, a mechanism-centred approach to theory development. We will adopt these steps to guide the process of realist synthesis: i) identify the research question, clarifying the scope of the review and formulating the initial programme theory (ii) conducting background searches in PubMed, PsycINFO, Africa-Wide Information, African.

Index Medicus (AIM), CINAHL and Scopus databases, and grey literature (iii) appraising the quality of studies and data extraction and (iv) synthesising data.

**Registration:**

The review is part of a realist evaluation exploring mental health policy implementation in LMICs and is registered under PROSPERO (registration number: CRD42024580312). Findings will inform the development of initial mental health policy implementation programme theories explaining why and how mental health policy implementation in LMICs works.

## Introduction

Mental health policy and plans are key instruments that governments require to address mental health needs at population level. Mental health policies often cover mental health promotion, prevention, and treatment of mental health conditions as well as mental health condition recovery. While mental health policies set out the values, principles and objectives that are organised to improve mental health and lessen the burden of mental health conditions in a population, plans set out the practical steps to realise these values, principles and objectives [1].

Successful implementation of these policies and plans can be supported by effective monitoring and evaluation activities. Based on World Health Organization survey responses, the percentage of countries in the low, lower middle and upper middle income groups that have no indicators or targets to monitor mental health policy implementation are 13%, 25% and 24%, respectively [1]. For those countries that have attempted to assess the success of their mental health policy, their monitoring and evaluation activities show varied outcomes with some countries having superior progress in mental health policy implementation than others. For example, from the same survey, there is a low uptake of human rights instruments to complement mental health policies and plans: 3% of countries in the low-income group, 14% in the lower-middle income group and 32% of countries in the upper-middle income group have mental health policies/plans that are in the process of implementation and fully compliant with human rights instruments [1].

There are several factors necessary for mental health policy implementation at national level. For instance, political will and good governance must be present. This is enabled by stakeholders, including mental health service users and other interest groups identifying priority areas and implementing the appropriate policies [2,3]. Creating an enabling organisational culture and ensuring accountability through role clarity of all stakeholders is vital for governance [4–6]. Good policy design should reflect planning for policy implementation from the policy development stage [3,6]. This ensures that the planned objectives are realistic and measurable so that when the plan is implemented, it gives the desired results. Effective and appropriate distribution of resources facilitates policy implementation. Ensuring that there is availability of resources and performing activities such as training on how the resources should be applied to the context, promotes efficient and effective use of these resources in mental health care [7]. Health providers should also have good systems that manage mental health service users health data [7] and programme implementation indicators for ongoing monitoring and evaluation [3].

However, low intersectoral collaboration, limited research, and financial and human resource constraints often underpin failed implementation efforts [8–10]. Obtaining more evidence on mental health policy implementation strategies, processes and outcomes is necessary to ensure successful mental health policy implementation in LMICs. LMICs are defined as countries with a gross national income (GNI) per capital which is less than USD13 205. Globally, 136 out of 217 countries (63%) fall into this category [11] which accounts for 6,62 billion of the world’s population. In LMICs, 48% of the people live in rural areas [12].

### Policy implementation strategies

Several mental health policy implementation strategies have been employed in different settings and countries worldwide. In this review, policy implementation strategies refer to techniques used to bring policy into practice, and approaches (‘route’ of implementation) followed to exercise control over various aspects of the policy implementation process. In implementation science, these implementation strategies are considered as the methods or techniques used to enhance the adoption, implementation, and sustainability of a policy. By identifying and classifying 73 implementation strategies,[13] provided conceptual clarity, relevance, and comprehensiveness of implementation strategies that can be used in isolation or combination in implementation research and practice.

#### Policy implementation techniques.

Policy implementation techniques are applied by governments to achieve their public policy objectives [14–16]. These include but are not limited to access new funding, change record systems, conduct ongoing training, identify and prepare champions and develop educational materials [13]. Although numerous typologies exist in the literature to classify policy implementation techniques, the Policy Ecology Framework has recently been updated specifically for characterising mental health policy implementation and is highly relevant for this review. The Policy Ecology Framework is for identifying and categorising mental health policy implementation techniques across four contextual domains. These include: the health provider organisation context, which includes mental health service users interacting with the organisation; the agency context, the political context and the social context. The agency context refers to the local or state bodies that provide oversight to the health provider organisation; whilst the political context refers to legislation and advocacy work that would enable mental health policy implementation and the social context are the structural and social factors that influence access to mental health services [17].

#### Policy implementation approaches.

Policy implementation approaches are the ‘routes’ of implementation, namely top-down, bottom-up or hybrid. This aspect of policy implementation strategy shows the influence of stakeholders on each other; and the autonomy which stakeholders possess in following or not following the stipulated ‘rules’. The top-down approach has policy-makers responsible for formulating the policy and policy implementers implementing the policy [16]. Bottom-up policy implementation involves policy implementers at local level who use their discretionary power to implement policies [16,18–20]. The hybrid policy implementation model is seen as a process of bargaining and transformation through blending the top-down and bottom-up approaches [21].

Mental health policy implementation within the healthcare system in many LMICs is described as top-down whereby policies are formulated at the national level and implemented at sub-national levels [22,23]. The bottom-up approach is described in other health policy implementation in community settings such as provision of primary health care services by different front-line workers. These include community health workers in Brazil [24], nurses in Kenyan and South African clinics, and environmental health officials in Ghana [25]. An example of hybrid policy implementation is proposed in the guidelines for national governments and their partners to design, implement and sustain effective and cost-effective HIV, TB and malaria community health worker programmes [26].

### Mental health policy implementation considerations for our review

Our review is situated at the policy implementation stage of the policy process, where the suggested policy solutions proposed by stakeholders at agenda setting and policy formulation stages are taken into action (or not) [16,27]. Due to a limited budget, the focus is mental health policy implementation at national level and the key stakeholders are policy makers and policy implementers at national level. While a recent systematic review has been conducted on methods and tools used to assess mental health policy and plan implementation [28], literature is lacking on the nuances of interactions between implementation strategies and implementation contexts, and how both positive and negative implementation outcomes are realised in LMIC settings. To address this gap and offer a fresh perspective, we will use a theory driven approach, namely realist review methodology, to explore and explain how and why the application of specified policy techniques and the application of top-down, bottom-up and hybrid approaches by the different policy stakeholders may result in (un)successful mental health policy implementation in LMICs. To this end, we aim to answer the following question: ‘How, why and under what circumstances is national mental health policy implemented in LMICs?’

### Aim and objectives

The aim of this study is to develop a realist review protocol to explain how, why, for whom, and under what health system conditions national mental health policy implementation works or not in LMICs.

The objectives of this study are: (1) to formulate an initial rough programme theory of how and why mental health policy implementation strategies work in LMICs; (2) to present the methods for conducting a realist review on mental health policy implementation in LMICs.

## Methods and analysis

The Preferred Reporting Items for Systematic Reviews and Meta-Analyses Protocols (PRISMA-P) 2015 checklist assisted development of this protocol [29]. A completed version is available as supplementary file 1.

### Realist review methodology

A realist review or synthesis is a theory-driven review informed by the realist philosophy of science [30]. It is aimed at formulating and testing (confirming, refuting or refining) initial programme theories to explain how, why, for whom and under what contexts a programme, intervention, or policy works or does not. The realist philosophy of science’s notion of causality suggests that entities existing within an open system, such as health systems, have causal effects on each other, where certain contextual factors (C), including the people involved and the sub-systems around them, trigger certain mechanisms (M) that in turn generate various outcomes (O) [30,31]. This implies that within a programme, including policy, an interplay of interactions within the prevailing circumstances and different actors result in intended or unintended consequences [30,31]. We provide definitions of context, mechanisms and outcomes in Table 1. Consequently, theory formulation in realist reviews is achieved through the context-mechanism-outcome (CMO) heuristic [32].

**Table 1:** Definitions of context, mechanisms and outcomes.

Context refers to the observable features such as space, place, people, things; or the relational and dynamic features that shape mechanisms through which an intervention works [33]. It is also understood as ‘an irreducible set of factors influencing when and how an intervention is delivered and how mechanisms are triggered’ [34].
**Mechanisms** as applied to the realist philosophy of science refer to psychological, social, cultural or organisational constructs that describe stakeholders’ attitudes, feelings or thoughts towards a programme [35]. This implies that they are hidden and need to be inferred from observable data [36] of stakeholders’ response to the resources available in the complex environment [37,38]. Mechanisms are not static as they are context sensitive, evolving within an open space-time and social system of relationships [36,38].
**Outcomes** are categorised as: *Patient-level* and *health service provision* outcomes are ‘the results of care in terms of patients’ health over time’ [39] and are placed in three tiers: patient health status achieved, process of recovery and sustainability of health. These speak to survival, time required to recover and degree of health maintained, respectively [39]*.* *Implementation outcomes* precede health service provision and health service use outcomes [40]. Eight domains are widely used in implementation studies, namely: acceptability, adoption, appropriateness, feasibility, fidelity, implementation cost, penetration, and sustainability [40]. Whitsel, Honeycutt [41] provide three additional domains namely, effectiveness, unintended consequences, and monitoring

A realist review starts with an initial rough theory based on substantive theories in the field of policy implementation and other relevant theoretical literature. In the review process, the initial theory is tested and consolidated through the search of secondary evidence to improve our understanding of the phenomenon [42–51] including health policies [52].

### Designing the review

**Fig 1:**
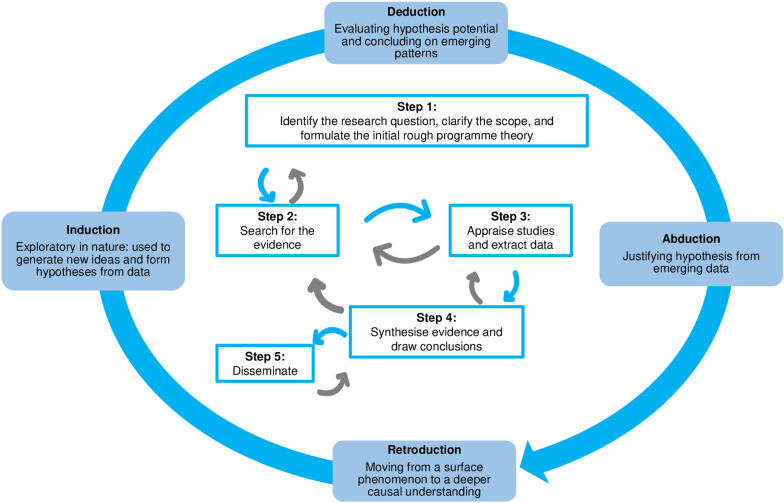
Steps for conducting the realist review *(adapted from [*55*]).*

The methods for our review will follow a non-linear realist synthesis design, based on the work of Pawson et al. (2004) with adaptations used in healthcare and education settings [53,54]. The following steps will be followed: (1) clarifying the scope of the review and formulating the initial programme theory, (2) conducting the processes of searching for relevant evidence to refine relevant theory, (3) appraising the quality of studies and data extraction using an eclectic and iterative approach, (4) data synthesis and (5) disseminating findings when the review is completed. This approach is depicted in Fig 1 below, from the work of Dada, Dalkin [55]. At each stage, hypotheses are developed through a process of induction, deduction, abduction and retroduction. These terms are explained further in Table 3 below.

#### Step 1: Identifying the question, clarifying the scope of the review, and formulate the initial programme theory.

**Fig 2:**
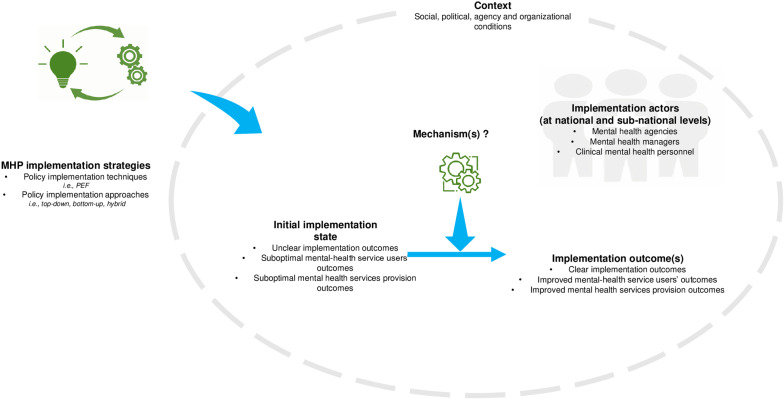
Framework for development of initial programme theory.

The review will be guided by the question, “How, why and under what circumstances is national mental health policy implemented in LMICs?” The scope of the review will be defined through conducting background searches followed by purposive searches. In realist review, this process is iterative and defining the scope can continue into the synthesis stage, whereby the explanatory nature of the review can be clearly refined through identifying the appropriate strategy or strategies [54,56]. For this review development of an initial theory will be guided by application of the Policy Ecology Framework. The domains employed within the Policy Ecology Framework largely examines the relation between the health organisation and the regulatory organisations within socio-political contexts [17] and our adaptation of the framework will delve deeper to consider unpacking mechanisms and how they are triggered by each contextual domain over time and setting (Fig 2). We postulate that if mental health policy in LMICs is developed at national level and implemented at subnational levels (i.e., provincial and district levels) using implementation strategies that promote/embody functional stakeholder engagements within diverse cultural, social, political and economic contexts, then favourable implementation, health service provision and mental health service users outcomes are achieved*.*

#### Step 2: Search for the evidence.

The initial theory and the aim of this review will guide the background search. Three background searches will be conducted to broaden our understanding of mental health policy implementation in LMICs. The first background search will review literature on policy implementation theories. The second background search will review qualitative, quantitative, and mixed methods studies on national or provincial mental health policy implementation strategies and plans in LMICs, whilst the third background search will be on available grey literature such as existing reports, policies and policy briefs on mental health policy implementation. In countries which do not use ‘province’, the equivalent term will be used. Studies that do not explicitly define the nature of the implementation plans and strategies will also be included. For grey literature, reported data will be searched which includes existing reports, policies, briefings, conference proceedings on mental health policy implementation in LMICs or from various stakeholders such as policy-makers and implementers, leaders of mental health advocacy groups or non-governmental organisations, health managers and coordinators.

#### Inclusion criteria.

Inclusion criteria only are recommended in the realist design because criteria are refined in the light of emerging data [56]. These are outlined in Table 2.

**Table 2:** Inclusion criteria.

***Design:*** literature describing national mental health policy implementation and/or implementation strategies CMOs implicitly and explicitly • Methodological papers explaining theory, frameworks or models on health policy implementation and implementation strategies • Empirical studies employing qualitative, quantitative or mixed methods • Grey literature such as existing reports, policies, policy briefs, or conference proceedings on mental health policy implementation
***Population***: mental health policy-makers and implementers at national or provincial level • Examples of these stakeholders include mental health national directorate, government mental health advocacy groups or non-governmental organisations, health managers and coordinators
***Setting***: low- and middle-income countries (LMICs) • Implementation at national and/or provincial levels ◦ In countries which do not use ‘province’ to describe a geographical or administrative region at sub-national level, the equivalent term will be used.
Other: • English language used for the write-up • Publications from 2001 to present. ◦ Publication date restrictions may shift from the selected databases due to hand searches and methodological papers

Following the background searches, we will also conduct purposive searches, which are conducted in realist reviews to iteratively guide the process of testing and refining programme theories [53,56]. Prior publications indexed on six online databases (PubMed, PsycINFO, Africa-Wide Information, African Index Medicus (AIM), CINAHL and Scopus) will be reviewed by a ‘berry-picking approach’ that supports the idea that search queries are ‘divergent and iterative, adaptable as emerging information is available’ [57]. This will be followed by hand-search of important citations and authors from articles extracted in databases [53].

The search for studies will be restricted to studies published in English from 2001 to date; as the first World Health Report on mental health was published in 2001 [58]. However, as there will be review of literature on policy implementation theories and hand searches, some references from other databases and prior to 2001 may be included.

The background searches will be analysed and synthesised into broad categories to inform the purposive searches. Another cycle on database, hand and grey literature searches will be repeated for the purposive searches. The inclusion criteria for purposive searches will be determined after the purposive search categories are defined. A list of theories will be generated whilst conducting the background and purposive searches and it will be shortened into a final list to determine the rough initial programme theories on mental health policy implementation in LMICs. For background and purposive databases searches, deduplication of records will occur. As this is a review on existing literature, no contact will be made with study authors for acquiring additional data. A draft search strategy for PubMed is provided in supplementary file 2. Further assistance will be sought from a librarian experienced in health-research reviews to allow for usage of the most relevant search terms and for the review to be methodologically sound.

#### Step 3: Appraise studies and extract data.

The eligibility criteria for appraising studies in realist studies must be ‘relevant and good enough’ for the synthesis. Relevance in realist review is about whether it addresses the theory being tested. Good enough speaks to rigour and richness. Rigour is whether a particular inference drawn by the original researcher has sufficient weight to make a methodologically credible contribution to the test of a particular intervention theory [55,56]. Richness is whether the studies clearly described theories and concepts, or describe the given programme [55]. In this review, any studies and grey literature that describe the context of policy implementation theories and mental health policy implementation plans and strategies in LMICs, explore mechanisms and outcomes, and are in alignment with the inclusion criteria and search terms will be appraised. We will use a flow diagram for realist reviews [59] to show the process of selecting studies (supplementary file 3).

Two people (Matima and Laurenzi) will independently review the quality of the data. Both Matima and Laurenzi have experience and background in health policy and systems research; particularly focusing on high burden diseases in the African context. Laurenzi has worked in adolescent and maternal mental health; and Matima is an early career researcher in diseases of high burden such as HIV and mental health conditions. To ensure reflexivity throughout Matima and Laurenzi will take independent notes of their assumptions during the review process and discuss these during joint review sessions. Where necessary, the two reviewers will also consult for further guidance on their notes and any issues that may arise in conducting the review with Lund and van der Westhuizen, experts in mental health implementation in LMICs; and Mukumbang, an expert in realist review methodology in healthcare. The quality of data will be screened in three cycles to explain, refine, or refute an initial programme theory. A ‘yes/no’ coding will be applied to each review cycle where ‘yes’ is if they meet the eligibility criterion and if not, ‘no’. At each cycle, a pass in screening will be ‘relevant and good enough’ to move to the next cycle for further review. A pass will entail having a ‘yes’ on a combination of two or more eligibility criteria for context, mechanisms or outcomes for the first two cycles; and for the third cycle, must make a credible contribution to development of initial theories. First, abstracts, second, titles for studies and grey literature that do not have abstracts, and third full-text will be screened for eligibility against the eligibility criteria. Articles relating to the same study will be linked. Before moving to the next review cycle, reviewers will agree on the final studies to include for further analysis. A flow chart will be developed that gives the databases and grey literature searched, the number of records found, deduplicated, excluded and the final studies for the review. The detailed process for data appraisal is described in the “Relevant and good enough procedure” tool (supplementary file 4).

#### Step 4: Synthesise evidence and draw conclusions.

In realist studies, data extraction and synthesis is guided by retroductive theorizing [31,60]. Data extraction forms will be created to extract data. Forms may be useful to sift, sort and annotate primary source data [54] and they consist of different lists of questions for the completion of different sections for the selected sources [56]. In this study, an initial version of the data extraction form adapted from multiple sources [61–63] will be used for all searches. Specific questions will be applied to different data sources (supplementary file 5) and collated data from the forms will be presented in one spreadsheet. Both reviewers will pilot the forms and as searches will be broad and repetitive, the forms will be adjusted where relevant. Once working versions of the form are agreed upon, one reviewer will extract the data and another will review it.

Both quantitative and qualitative data are synthesised to refine programme theory [54]. Further methodological recommendations exist in literature on synthesising qualitative evidence and a realist approach to thematic analysis [64,65] will be applied to any qualitative studies that may form part of the data collected for the review. Thematic analysis of qualitative studies is stratified according to three themes: experiential, inferential and dispositional themes. Experiential themes follow deductive thinking; inferential follow inductive and abductive thinking; and dispositional themes follow retroductive thinking [64].

To develop a final model from the synthesised data, plausible initial theories will contain a CMO configuration stemming from the dispositional themes. While the ‘dispositional-inferential-experiential’ format may be applied to summarise CMO configurations for qualitative studies [64], the ‘if (context) then (outcome) because (mechanism)’ formulation has commonly been used to mixed data [66–68] and will be adapted to synthesise the data in this review. The formulation of statements aid the unpacking of CMO configurations in the data as three elements (CMO) or as two-elements (CM or CO or MO) [65]. Understanding these terms is key to undertaking a realist review and their meanings are further described in Table 3.

**Table 3:** Descriptions of key realist synthesis terms for this review.

Conducting a realist synthesis is shaped by the processes of induction, deduction, abduction and retroduction.
• ***Induction*** begins with observations and moves to making broad generalizations and predictions. In other words, we start with what we know about an observation and move to what we do not know [60].
• ***Deduction*** begins with an initial theory, assumption or perspective and then applies the theory, assumption or perspective to the observations in order to further elucidate the topic of study [60].
• ***Abduction*** is justifying the more concrete theories or assumptions [60]
• ***Retroduction*** is the investigation of causal mechanisms and the conditions under which certain outcomes will or will not be realised [69] and is prominent at the data synthesis stage. It is also understood as the ‘empirical process of devising a theory’ [60] through identifying the latent mechanisms that characterise observable phenomena [60,64,70] ◦** Dispositional themes** are the ‘theories about the properties and powers that must exist in order to produce the phenomena being studied’ [64]. **Retroductive theorizing** (which derives from the word **retroduction**), is applied to generate dispositional themes [64].

## Dissemination

Reporting the review will be aligned with RAMESES publication standards for realist syntheses [36]. The review is part of a broader realist evaluation exploring mental health policy implementation in LMICs. The review will inform the initial theory that will be tested and consolidated later as a case study on South African mental health policy implementation. The review will be published as a journal article.

## Supporting information

S1 File PRISMA-P Checklist (DOCX)

S2 File PubMed Draft Search Strategy (PDF)

S3 File Flow Chart for realist review searches (DOCX)

S4 File Relevant and good enough screening procedure (DOCX)

S5 File Data extraction form for background searches (DOCX)
