# Supplementary material for: Mental health policy implementation in low- and middle-income countries: a realist review protocol
Source: PLoS One. 2025 Mar 25;20(3):e0320420. doi: 10.1371/journal.pone.0320420 (PMC11936231; doi:10.1371/journal.pone.0320420)
Supplement: S2 File — (PDF) [file pone.0320420.s002.pdf]

Supplementary File 2: PubMed Draft Search Strategy

| Search number | Query | Search Details | Results |
|---|---|---|---|
| #1 | Mental Health Services[MeSH Terms] | "mental health services"[MeSH Terms] | 108,571 |
| #2 | Mental Health Policy[Text Word] OR Mental Health Policies[Text Word] OR Mental Health Strategy[Text Word] OR Mental Health Strategies[Text Word] OR Mental Health Service[Text Word] OR Mental Health Services[Text Word] OR Mental Health Intervention[Text Word] OR Mental Health Interventions[Text Word] OR mental health plan[Text Word] OR mental health plans[Text Word] OR Mental Health Program[Text Word] OR Mental Health Programs[Text Word] | "mental health policy"[Text Word] OR "mental health policies"[Text Word] OR "mental health strategy"[Text Word] OR "mental health strategies"[Text Word] OR "mental health service"[Text Word] OR "mental health services"[Text Word] OR "mental health intervention"[Text Word] OR "mental health interventions"[Text Word] OR "mental health plan"[Text Word] OR "mental health plans"[Text Word] OR "mental health program"[Text Word] OR "mental health programs"[Text Word] | 78,326 |
| #3 | Implementation Science OR Evidence-Based Practice OR Health Plan Implementation OR Program Evaluation[MeSH Terms] | "implementation science"[MeSH Terms] OR ("implementation"[All Fields] AND "science"[All Fields]) OR "implementation science"[All Fields] OR ("evidence based practice"[MeSH Terms] OR ("evidence based"[All Fields] AND "practice"[All Fields]) OR "evidence based practice"[All Fields] OR ("evidence"[All Fields] AND "based"[All Fields] AND "practice"[All Fields]) OR "evidence based practice"[All Fields]) OR ("health plan implementation"[MeSH Terms] OR ("health"[All Fields] AND "plan"[All Fields] AND "implementation"[All Fields]) OR "health plan implementation"[All Fields]) OR "program evaluation"[MeSH Terms] | 348,910 |
| #4 | Evaluation[Text Word] OR Implementation[Text Word] OR adoption[Text Word] OR Enforcement[Text Word] OR dissemination[Text Word] OR scale-up[Text Word] OR scale up[Text Word] OR scaling[Text Word] OR translational research[Text Word] OR evidence based practice[Text Word] OR evidence-based practice[Text Word] | "Evaluation"[Text Word] OR "Implementation"[Text Word] OR "adoption"[Text Word] OR "Enforcement"[Text Word] OR "dissemination"[Text Word] OR "scale-up"[Text Word] OR "scale-up"[Text Word] OR "scaling"[Text Word] OR "translational research"[Text Word] OR "evidence based practice"[Text Word] OR "evidence based practice"[Text Word] | 2,653,392 |
| #5 | #1 OR #2 | "mental health services"[MeSH Terms] OR "mental health policy"[Text Word] OR "mental health policies"[Text Word] OR "mental health strategy"[Text Word] OR "mental health strategies"[Text Word] OR "mental health service"[Text Word] OR "mental health services"[Text Word] OR "mental health intervention"[Text Word] OR "mental health interventions"[Text Word] OR "mental health plan"[Text Word] OR "mental health plans"[Text Word] OR "mental health program"[Text Word] OR "mental health programs"[Text Word] | 127,904 |
| #6 | #3 OR #4 | "implementation science"[MeSH Terms] OR ("Implementation"[All Fields] AND "science"[All Fields]) OR "implementation science"[All Fields] OR ("evidence based practice"[MeSH Terms] OR ("evidence-based"[All Fields] AND "practice"[All Fields]) OR "evidence based practice"[All Fields] OR ("evidence"[All Fields] AND "based"[All Fields] AND "practice"[All Fields]) OR "evidence based practice"[All Fields]) OR ("health plan implementation"[MeSH Terms] OR ("health"[All Fields] AND "plan"[All Fields] AND "Implementation"[All Fields]) OR "health plan implementation"[All Fields]) OR "program evaluation"[MeSH Terms] OR ("Evaluation"[Text Word] OR "Implementation"[Text Word] OR "adoption"[Text Word] OR "Enforcement"[Text Word] OR "dissemination"[Text Word] OR "scale-up"[Text Word] OR "scale-up"[Text Word] OR "scaling"[Text Word] OR "translational research"[Text Word] OR "evidence based practice"[Text Word] OR "evidence based practice"[Text Word]) | 2,794,649 |
| #7 | #5 AND #6 | ("mental health services"[MeSH Terms] OR ("mental health policy"[Text Word] OR "mental health policies"[Text Word] OR "mental health strategy"[Text Word] OR "mental health strategies"[Text Word] OR "mental health service"[Text Word] OR "mental health services"[Text Word] OR "mental health intervention"[Text Word] OR "mental health interventions"[Text Word] OR "mental health plan"[Text Word] OR "mental health plans"[Text Word] OR "mental health program"[Text Word] OR "mental health programs"[Text Word])) AND ("implementation science"[MeSH Terms] OR ("Implementation"[All Fields] AND "science"[All Fields]) OR "implementation science"[All Fields] OR ("evidence based practice"[MeSH Terms] OR ("evidence-based"[All Fields] AND "practice"[All Fields]) OR "evidence based practice"[All Fields] OR ("evidence"[All Fields] AND "based"[All Fields] AND "practice"[All Fields]) OR "evidence based practice"[All Fields]) OR ("health plan implementation"[MeSH Terms] OR ("Health"[All Fields] AND "plan"[All Fields] AND "Implementation"[All Fields]) OR "health plan implementation"[All Fields]) OR "program evaluation"[MeSH Terms] OR ("Evaluation"[Text Word] OR "Implementation"[Text Word] OR "adoption"[Text Word] OR "Enforcement"[Text Word] OR "dissemination"[Text Word] OR "scale-up"[Text Word] OR "scale-up"[Text Word] OR "scaling"[Text Word] OR "translational research"[Text Word] OR "evidence based practice"[Text Word] OR "evidence based practice"[Text Word])) | 24,737 |

| | | | |
|---|---|---|---|
| #8 | Timeline: 2001 to date | (("mental health services"[MeSH Terms] OR ("mental health policy"[Text Word] OR "mental health policies"[Text Word] OR "mental health strategy"[Text Word] OR "mental health strategies"[Text Word] OR "mental health service"[Text Word] OR "mental health services"[Text Word] OR "mental health intervention"[Text Word] OR "mental health interventions"[Text Word] OR "mental health plan"[Text Word] OR "mental health plans"[Text Word] OR "mental health program"[Text Word] OR "mental health programs"[Text Word])) AND ("implementation science"[MeSH Terms] OR ("Implementation"[All Fields] AND "science"[All Fields]) OR "implementation science"[All Fields] OR ("evidence based practice"[MeSH Terms] OR ("evidence-based"[All Fields] AND "practice"[All Fields]) OR "evidence based practice"[All Fields] OR ("evidence"[All Fields] AND "based"[All Fields] AND "practice"[All Fields]) OR "evidence based practice"[All Fields]) OR ("health plan implementation"[MeSH Terms] OR ("Health"[All Fields] AND "plan"[All Fields] AND "Implementation"[All Fields]) OR "health plan implementation"[All Fields]) OR "program evaluation"[MeSH Terms] OR ("Evaluation"[Text Word] OR "Implementation"[Text Word] OR "adoption"[Text Word] OR "Enforcement"[Text Word] OR "dissemination"[Text Word] OR "scale-up"[Text Word] OR "scale-up"[Text Word] OR "scaling"[Text Word] OR "translational research"[Text Word] OR "evidence based practice"[Text Word] OR "evidence based practice"[Text Word]))) AND (2001:2024[pdat]) | 20,483 |
| #9 | Species: Humans | (("mental health services"[MeSH Terms] OR ("mental health policy"[Text Word] OR "mental health policies"[Text Word] OR "mental health strategy"[Text Word] OR "mental health strategies"[Text Word] OR "mental health service"[Text Word] OR "mental health services"[Text Word] OR "mental health intervention"[Text Word] OR "mental health interventions"[Text Word] OR "mental health plan"[Text Word] OR "mental health plans"[Text Word] OR "mental health program"[Text Word] OR "mental health programs"[Text Word])) AND ("implementation science"[MeSH Terms] OR ("Implementation"[All Fields] AND "science"[All Fields]) OR "implementation science"[All Fields] OR ("evidence based practice"[MeSH Terms] OR ("evidence-based"[All Fields] AND "practice"[All Fields]) OR "evidence based practice"[All Fields] OR ("evidence"[All Fields] AND "based"[All Fields] AND "practice"[All Fields]) OR "evidence based practice"[All Fields]) OR ("health plan implementation"[MeSH Terms] OR ("Health"[All Fields] AND "plan"[All Fields] AND "Implementation"[All Fields]) OR "health plan implementation"[All Fields]) OR "program evaluation"[MeSH Terms] OR ("Evaluation"[Text Word] OR "Implementation"[Text Word] OR "adoption"[Text Word] OR "Enforcement"[Text Word] OR "dissemination"[Text Word] OR "scale-up"[Text Word] OR "scale-up"[Text Word] OR "scaling"[Text Word] OR "translational research"[Text Word] OR "evidence based practice"[Text Word] OR "evidence based practice"[Text Word]))) AND ((humans[Filter]) AND (2001:2024[pdat])) | 18,558 |
| #10 | Article Language: English | (("mental health services"[MeSH Terms] OR ("mental health policy"[Text Word] OR "mental health policies"[Text Word] OR "mental health strategy"[Text Word] OR "mental health strategies"[Text Word] OR "mental health service"[Text Word] OR "mental health services"[Text Word] OR "mental health intervention"[Text Word] OR "mental health interventions"[Text Word] OR "mental health plan"[Text Word] OR "mental health plans"[Text Word] OR "mental health program"[Text Word] OR "mental health programs"[Text Word])) AND ("implementation science"[MeSH Terms] OR ("Implementation"[All Fields] AND "science"[All Fields]) OR "implementation science"[All Fields] OR ("evidence based practice"[MeSH Terms] OR ("evidence-based"[All Fields] AND "practice"[All Fields]) OR "evidence based practice"[All Fields] OR ("evidence"[All Fields] AND "based"[All Fields] AND "practice"[All Fields]) OR "evidence based practice"[All Fields]) OR ("health plan implementation"[MeSH Terms] OR ("Health"[All Fields] AND "plan"[All Fields] AND "Implementation"[All Fields]) OR "health plan implementation"[All Fields]) OR "program evaluation"[MeSH Terms] OR ("Evaluation"[Text Word] OR "Implementation"[Text Word] OR "adoption"[Text Word] OR "Enforcement"[Text Word] OR "dissemination"[Text Word] OR "scale-up"[Text Word] OR "scale-up"[Text Word] OR "scaling"[Text Word] OR "translational research"[Text Word] OR "evidence based practice"[Text Word] OR "evidence based practice"[Text Word]))) AND ((humans[Filter]) AND (english[Filter]) AND (2001:2024[pdat])) | 17,675 |
| #11 | Geographical limits: LMIC filter | ("deprived countries"[Text Word] OR "deprived population"[Text Word] OR "deprived populations"[Text Word] OR "developing countries"[Text Word] OR "developing country"[Text Word] OR "developing economies"[Text Word] OR "developing economy"[Text Word] OR "developing nation"[Text Word] OR "developing nations"[Text Word] OR "developing population"[Text Word] OR "developing populations"[Text Word] OR "developing world"[Text Word] OR "lami countries"[Text Word] OR "lami country"[Text Word] OR "less developed countries"[Text Word] OR "less developed country"[Text Word] OR "less developed economies"[Text Word] OR "less developed nation"[Text Word] OR "less developed nations"[Text Word] OR "less developed world"[Text Word] OR "lesser developed countries"[Text Word] OR "lesser developed nations"[Text Word] OR "LMIC"[Text Word] OR "LMICS"[Text Word] OR "low gdp"[Text Word] OR "low gnp"[Text Word] OR "low gross domestic"[Text Word] OR "low gross national"[Text Word] OR "low income countries"[Text Word] OR "low income country"[Text Word] OR "low income economies"[Text Word] OR "low income economy"[Text Word] OR "low income nations"[Text Word] OR "low income population"[Text Word] OR "low income populations"[Text Word] OR "lower gdp"[Text Word] OR "lower gross domestic"[Text Word] OR "lower income countries"[Text Word] OR "lower income country"[Text Word] OR "lower income nations"[Text Word] OR "lower income population"[Text Word] OR "lower income populations"[Text Word] OR "middle income countries"[Text Word] OR "middle income country"[Text Word] OR "middle income economies"[Text Word] OR "middle income nation"[Text Word] OR "middle income nations"[Text Word] OR "middle income population"[Text Word] OR "middle income populations"[Text Word] OR "poor countries"[Text Word] OR "poor country"[Text Word] OR "poor economies"[Text Word] OR "poor economy"[Text Word] OR "poor nation"[Text Word] OR "poor nations"[Text Word] OR "poor population"[Text Word] OR "poor populations"[Text Word] OR "poor world"[Text Word] OR "poorer countries"[Text Word] OR "poorer economies"[Text Word] OR "poorer economy"[Text Word] OR "poorer nations"[Text Word] OR "poorer population"[Text Word] OR "poorer populations"[Text Word] OR "third world"[Text Word] OR "transitional countries"[Text Word] OR "transitional country"[Text Word] OR "transitional economies"[Text Word] OR "transitional economy"[Text Word] OR "under developed countries"[Text Word] OR "under developed country"[Text Word] OR "under developed nations"[Text Word] OR "under developed world"[Text Word] OR "under served population"[Text Word] OR "under served populations"[Text Word] OR "underdeveloped countries"[Text Word] OR "underdeveloped country"[Text Word] OR "underdeveloped economies"[Text Word] OR "underdeveloped nations"[Text Word] OR "underdeveloped population"[Text Word] OR "underdeveloped world"[Text Word] OR "underserved countries"[Text Word] OR "underserved nations"[Text Word] OR "underserved population"[Text Word] OR "underserved populations"[Text Word] OR "Afghanistan"[Text Word] OR "Albania"[Text Word] OR "Algeria"[Text Word] OR "american samoa"[Text Word] OR "Angola"[Text Word] OR "Armenia"[Text Word] OR "Azerbaijan"[Text Word] OR "Bangladesh"[Text Word] OR "Belarus"[Text Word] OR "Byelarus"[Text Word] OR "Belorussia"[Text Word] OR "Belize"[Text Word] OR "Benin"[Text Word] OR "Bhutan"[Text Word] OR "Bolivia"[Text Word] OR "Bosnia"[Text Word] OR "Botswana"[Text Word] OR "Brazil"[Text Word] OR "Bulgaria"[Text Word] OR "Burma"[Text Word] OR "burkina faso"[Text Word] OR "Burundi"[Text Word] OR "cabo | 1,110,733 |

| | | | |
|---|---|---|---|
| | | verde"[Text Word] OR "cape verde"[Text Word] OR "Cambodia"[Text Word] OR "Cameroon"[Text Word] OR "central african republic"[Text Word] OR "Chad"[Text Word] OR "China"[Text Word] OR "Colombia"[Text Word] OR "Comoros"[Text Word] OR "Comores"[Text Word] OR "Comoro"[Text Word] OR "Congo"[Text Word] OR "costa rica"[Text Word] OR "cote d ivoire"[Text Word] OR "Cuba"[Text Word] OR "Djibouti"[Text Word] OR "Dominica"[Text Word] OR "dominican republic"[Text Word] OR "Ecuador"[Text Word] OR "Egypt"[Text Word] OR "el salvador"[Text Word] OR "equatorial guinea"[Text Word] OR "Eritrea"[Text Word] OR "Ethiopia"[Text Word] OR "Fiji"[Text Word] OR "Gabon"[Text Word] OR "Gambia"[Text Word] OR "Gaza"[Text Word] OR "Georgia"[Text Word] OR "georgia republic"[Text Word] OR "Ghana"[Text Word] OR "Grenada"[Text Word] OR "Grenadines"[Text Word] OR "Guatemala"[Text Word] OR "Guinea"[Text Word] OR "guinea bissau"[Text Word] OR "Guyana"[Text Word] OR "Haiti"[Text Word] OR "Herzegovina"[Text Word] OR "Hercegovina"[Text Word] OR "Honduras"[Text Word] OR "India"[Text Word] OR "Indonesia"[Text Word] OR "Iran"[Text Word] OR "Iraq"[Text Word] OR "ivory coast"[Text Word] OR "Jamaica"[Text Word] OR "Jordan"[Text Word] OR "Kazakhstan"[Text Word] OR "Kenya"[Text Word] OR "Kiribati"[Text Word] OR "democratic people s republic of korea"[Text Word] OR "Kosovo"[Text Word] OR "Kyrgyz"[Text Word] OR "Kirghizia"[Text Word] OR "Kirghiz"[Text Word] OR "Kyrgyzstan"[Text Word] OR "lao pdr"[Text Word] OR "Laos"[Text Word] OR "Lebanon"[Text Word] OR "Lesotho"[Text Word] OR "Liberia"[Text Word] OR "Libya"[Text Word] OR "Macedonia"[Text Word] OR "Madagascar"[Text Word] OR "Malawi"[Text Word] OR "Malay"[Text Word] OR "Malaya"[Text Word] OR "Malaysia"[Text Word] OR "Maldives"[Text Word] OR "Mali"[Text Word] OR "marshall islands"[Text Word] OR "Mauritania"[Text Word] OR "Mauritius"[Text Word] OR "Mexico"[Text Word] OR "Micronesia"[Text Word] OR "Moldova"[Text Word] OR "Mongolia"[Text Word] OR "Montenegro"[Text Word] OR "Morocco"[Text Word] OR "Mozambique"[Text Word] OR "Myanmar"[Text Word] OR "Namibia"[Text Word] OR "Nepal"[Text Word] OR "Nicaragua"[Text Word] OR "Niger"[Text Word] OR "Nigeria"[Text Word] OR "Pakistan"[Text Word] OR "Palau"[Text Word] OR "papua new guinea"[Text Word] OR "Paraguay"[Text Word] OR "Peru"[Text Word] OR "Philippines"[Text Word] OR "Principe"[Text Word] OR "Romania"[Text Word] OR "Ruanda"[Text Word] OR "Rwanda"[Text Word] OR "Samoa"[Text Word] OR "sao tome"[Text Word] OR "Senegal"[Text Word] OR "Serbia"[Text Word] OR "sierra leone"[Text Word] OR "solomon islands"[Text Word] OR "Somalia"[Text Word] OR "south africa"[Text Word] OR "south sudan"[Text Word] OR "sri lanka"[Text Word] OR "st lucia"[Text Word] OR "st vincent"[Text Word] OR "Sudan"[Text Word] OR "Surinam"[Text Word] OR "Suriname"[Text Word] OR "Swaziland"[Text Word] OR "Syria"[Text Word] OR "syrian arab republic"[Text Word] OR "Tajikistan"[Text Word] OR "Tadzhikistan"[Text Word] OR "Tajikistan"[Text Word] OR "Tadzhik"[Text Word] OR "Tanzania"[Text Word] OR "Thailand"[Text Word] OR "Timor"[Text Word] OR "Togo"[Text Word] OR "Tonga"[Text Word] OR "Tunisia"[Text Word] OR "Turkey"[Text Word] OR "Turkmen"[Text Word] OR "Turkmenistan"[Text Word] OR "Tuvalu"[Text Word] OR "Uganda"[Text Word] OR "Ukraine"[Text Word] OR "Uzbek"[Text Word] OR "Uzbekistan"[Text Word] OR "Vanuatu"[Text Word] OR "Venezuela"[Text Word] OR "Vietnam"[Text Word] OR "west bank"[Text Word] OR "Yemen"[Text Word] OR "Zambia"[Text Word] OR "Zimbabwe"[Text Word]) AND ((humans[Filter]) AND (english[Filter])) | |
| #12 | #10 AND #11 | (("mental health services"[MeSH Terms] OR ("mental health policy"[Text Word] OR "mental health policies"[Text Word] OR "mental health strategy"[Text Word] OR "mental health strategies"[Text Word] OR "mental health service"[Text Word] OR "mental health services"[Text Word] OR "mental health intervention"[Text Word] OR "mental health interventions"[Text Word] OR "mental health plan"[Text Word] OR "mental health plans"[Text Word] OR "mental health program"[Text Word] OR "mental health programs"[Text Word])) AND ("implementation science"[MeSH Terms] OR ("Implementation"[All Fields] AND "science"[All Fields]) OR "implementation science"[All Fields] OR ("evidence based practice"[MeSH Terms] OR ("evidence-based"[All Fields] AND "practice"[All Fields]) OR "evidence based practice"[All Fields] OR ("evidence"[All Fields] AND "based"[All Fields] AND "practice"[All Fields]) OR "evidence based practice"[All Fields]) OR ("health plan implementation"[MeSH Terms] OR ("Health"[All Fields] AND "plan"[All Fields] AND "Implementation"[All Fields]) OR "health plan implementation"[All Fields]) OR "program evaluation"[MeSH Terms] OR ("Evaluation"[Text Word] OR "Implementation"[Text Word] OR "adoption"[Text Word] OR "Enforcement"[Text Word] OR "dissemination"[Text Word] OR "scale-up"[Text Word] OR "scale-up"[Text Word] OR "scaling"[Text Word] OR "translational research"[Text Word] OR "evidence based practice"[Text Word] OR "evidence based practice"[Text Word])) AND ("humans"[MeSH Terms] AND "english"[Language] AND 2001/01/01:2024/12/31[Date - Publication]) AND (("deprived countries"[Text Word] OR "deprived population"[Text Word] OR "deprived populations"[Text Word] OR "developing countries"[Text Word] OR "developing country"[Text Word] OR "developing economies"[Text Word] OR "developing economy"[Text Word] OR "developing nation"[Text Word] OR "developing nations"[Text Word] OR "developing population"[Text Word] OR "developing populations"[Text Word] OR "developing world"[Text Word] OR "lami countries"[Text Word] OR "lami country"[Text Word] OR "less developed countries"[Text Word] OR "less developed country"[Text Word] OR "less developed economies"[Text Word] OR "less developed nation"[Text Word] OR "less developed nations"[Text Word] OR "less developed world"[Text Word] OR "lesser developed countries"[Text Word] OR "lesser developed nations"[Text Word] OR "LMIC"[Text Word] OR "LMICS"[Text Word] OR "low gdp"[Text Word] OR "low gnp"[Text Word] OR "low gross domestic"[Text Word] OR "low gross national"[Text Word] OR "low income countries"[Text Word] OR "low income country"[Text Word] OR "low income economies"[Text Word] OR "low income economy"[Text Word] OR "low income nations"[Text Word] OR "low income population"[Text Word] OR "low income populations"[Text Word] OR "lower gdp"[Text Word] OR "lower gross domestic"[Text Word] OR "lower income countries"[Text Word] OR "lower income country"[Text Word] OR "lower income nations"[Text Word] OR "lower income population"[Text Word] OR "lower income populations"[Text Word] OR "middle income countries"[Text Word] OR "middle income country"[Text Word] OR "middle income economies"[Text Word] OR "middle income nation"[Text Word] OR "middle income nations"[Text Word] OR "middle income population"[Text Word] OR "middle income populations"[Text Word] OR "poor countries"[Text Word] OR "poor country"[Text Word] OR "poor economies"[Text Word] OR "poor economy"[Text Word] OR "poor nation"[Text Word] OR "poor nations"[Text Word] OR "poor population"[Text Word] OR "poor populations"[Text Word] OR "poor world"[Text Word] OR "poorer countries"[Text Word] OR "poorer economies"[Text Word] OR "poorer economy"[Text Word] OR "poorer nations"[Text Word] OR "poorer population"[Text Word] OR "poorer populations"[Text Word] OR "third world"[Text Word] OR "transitional countries"[Text Word] OR "transitional country"[Text Word] OR "transitional economies"[Text Word] OR "transitional economy"[Text Word] OR "under developed countries"[Text Word] OR "under developed country"[Text Word] OR "under developed nations"[Text Word] OR "under developed world"[Text Word] OR "under served population"[Text Word] OR "under served populations"[Text Word] OR "underdeveloped countries"[Text Word] OR "underdeveloped country"[Text Word] OR "underdeveloped economies"[Text Word] OR "underdeveloped nations"[Text Word] OR "underdeveloped population"[Text Word] OR "underdeveloped world"[Text Word] OR "underserved countries"[Text Word] OR "underserved nations"[Text Word] OR "underserved population"[Text Word] OR "underserved populations"[Text Word] OR ("Afghanistan"[Text Word] OR "Albania"[Text Word] OR "Algeria"[Text Word] OR "american samoa"[Text Word] OR "Angola"[Text Word] OR "Armenia"[Text Word] OR "Azerbaijan"[Text Word] OR "Bangladesh"[Text Word] OR "Belarus"[Text Word] OR "Byelarus"[Text Word] OR "Belorussia"[Text Word] OR "Belize"[Text Word] OR "Benin"[Text Word] OR "Bhutan"[Text Word] OR "Bolivia"[Text Word] OR "Bosnia"[Text Word] OR | 2,448 |

| | | | | |
|---|---|---|---|---|
| | | | "Botswana"[Text Word] OR "Brazil"[Text Word] OR "Bulgaria"[Text Word] OR "Burma"[Text Word] OR "burkina faso"[Text Word] OR "Burundi"[Text Word] OR "cabo verde"[Text Word] OR "cape verde"[Text Word] OR "Cambodia"[Text Word] OR "Cameroon"[Text Word] OR "central african republic"[Text Word] OR "Chad"[Text Word] OR "China"[Text Word] OR "Colombia"[Text Word] OR "Comoros"[Text Word] OR "Comores"[Text Word] OR "Comoro"[Text Word] OR "Congo"[Text Word] OR "costa rica"[Text Word] OR "cote d ivoire"[Text Word] OR "Cuba"[Text Word] OR "Djibouti"[Text Word] OR "Dominica"[Text Word] OR "dominican republic"[Text Word] OR "Ecuador"[Text Word] OR "Egypt"[Text Word] OR "el salvador"[Text Word] OR "equatorial guinea"[Text Word] OR "Eritrea"[Text Word] OR "Ethiopia"[Text Word] OR "Fiji"[Text Word] OR "Gabon"[Text Word] OR "Gambia"[Text Word] OR "Gaza"[Text Word] OR "Georgia"[Text Word] OR "georgia republic"[Text Word] OR "Ghana"[Text Word] OR "Grenada"[Text Word] OR "Grenadines"[Text Word] OR "Guatemala"[Text Word] OR "Guinea"[Text Word] OR "guinea bissau"[Text Word] OR "Guyana"[Text Word] OR "Haiti"[Text Word] OR "Herzegovina"[Text Word] OR "Hercegovina"[Text Word] OR "Honduras"[Text Word] OR "India"[Text Word] OR "Indonesia"[Text Word] OR "Iran"[Text Word] OR "Iraq"[Text Word] OR "ivory coast"[Text Word] OR "Jamaica"[Text Word] OR "Jordan"[Text Word] OR "Kazakhstan"[Text Word] OR "Kenya"[Text Word] OR "Kiribati"[Text Word] OR "democratic people s republic of korea"[Text Word] OR "Kosovo"[Text Word] OR "Kyrgyz"[Text Word] OR "Kirghizia"[Text Word] OR "Kirghiz"[Text Word] OR "Kyrgyzstan"[Text Word] OR "lao pdr"[Text Word] OR "Laos"[Text Word] OR "Lebanon"[Text Word] OR "Lesotho"[Text Word] OR "Liberia"[Text Word] OR "Libya"[Text Word] OR "Macedonia"[Text Word] OR "Madagascar"[Text Word] OR "Malawi"[Text Word] OR "Malay"[Text Word] OR "Malaya"[Text Word] OR "Malaysia"[Text Word] OR "Maldives"[Text Word] OR "Mali"[Text Word] OR "marshall islands"[Text Word] OR "Mauritania"[Text Word] OR "Mauritius"[Text Word] OR "Mexico"[Text Word] OR "Micronesia"[Text Word] OR "Moldova"[Text Word] OR "Mongolia"[Text Word] OR "Montenegro"[Text Word] OR "Morocco"[Text Word] OR "Mozambique"[Text Word] OR "Myanmar"[Text Word] OR "Namibia"[Text Word] OR "Nepal"[Text Word] OR "Nicaragua"[Text Word] OR "Niger"[Text Word] OR "Nigeria"[Text Word] OR "Pakistan"[Text Word] OR "Palau"[Text Word] OR "papua new guinea"[Text Word] OR "Paraguay"[Text Word] OR "Peru"[Text Word] OR "Philippines"[Text Word] OR "Principe"[Text Word] OR "Romania"[Text Word] OR "Ruanda"[Text Word] OR "Rwanda"[Text Word] OR "Samoa"[Text Word] OR "sao tome"[Text Word] OR "Senegal"[Text Word] OR "Serbia"[Text Word] OR "sierra leone"[Text Word] OR "solomon islands"[Text Word] OR "Somalia"[Text Word] OR "south africa"[Text Word] OR "south sudan"[Text Word] OR "sri lanka"[Text Word] OR "st lucia"[Text Word] OR "st vincent"[Text Word] OR "Sudan"[Text Word] OR "Surinam"[Text Word] OR "Suriname"[Text Word] OR "Swaziland"[Text Word] OR "Syria"[Text Word] OR "syrian arab republic"[Text Word] OR "Tajikistan"[Text Word] OR "Tadzhikistan"[Text Word] OR "Tajikistan"[Text Word] OR "Tadzhik"[Text Word] OR "Tanzania"[Text Word] OR "Thailand"[Text Word] OR "Timor"[Text Word] OR "Togo"[Text Word] OR "Tonga"[Text Word] OR "Tunisia"[Text Word] OR "Turkey"[Text Word] OR "Turkmen"[Text Word] OR "Turkmenistan"[Text Word] OR "Tuvalu"[Text Word] OR "Uganda"[Text Word] OR "Ukraine"[Text Word] OR "Uzbek"[Text Word] OR "Uzbekistan"[Text Word] OR "Vanuatu"[Text Word] OR "Venezuela"[Text Word] OR "Vietnam"[Text Word] OR "west bank"[Text Word] OR "Yemen"[Text Word] OR "Zambia"[Text Word] OR "Zimbabwe"[Text Word])) AND ("humans"[MeSH Terms] AND "english"[Language]))) AND ((humans[Filter]) AND (english[Filter])) | |
