# Supplementary material for: Mental health policy implementation in low- and middle-income countries: a realist review protocol
Source: PLoS One. 2025 Mar 25;20(3):e0320420. doi: 10.1371/journal.pone.0320420 (PMC11936231; doi:10.1371/journal.pone.0320420)
Supplement: S3 File — (DOCX) [file pone.0320420.s003.docx]

**Iterative search 1**

**(any study meeting broad criteria)**

**Initial search**

**(any study meeting broad criteria)**

**Iterative search 2, etc**

**(any study meeting broad criteria)**

Records identified through database and grey literature searches: ***(N* = )**

Database 1 (n= )

Database 2 (n= )

etc.

Grey literature (n =)

Records identified through forward-backwards citations and hand searches of key journals:

***(N* = )**

Records identified through forward-backwards citations and hand searches of key journals:

***(N* = )**

Records identified through database and grey literature searches: ***(N* = )**

Database 1 (n= )

Database 2 (n= )

etc.

Grey literature (n =)

Records identified through forward-backwards citations and hand searches of key journals:

***(N* = )**

Records identified through database and grey literature searches: ***(N* = )**

Database 1 (n= )

Database 2 (n= )

etc.

Grey literature (n =)

**Identification**

Duplicate records excluded (n = )

Duplicate records excluded (n = )

Duplicate records excluded (n = )

Records remaining after deduplication screened for relevance: title and abstract screening

(n = )

Records remaining after deduplication screened for relevance: title and abstract screening

(n = )

Records remaining after deduplication screened for relevance: title and abstract screening

(n = )

Records excluded based on title not being relevant to topic of interest:

(n = )

Records excluded based on title not being relevant to topic of interest:

(n = )

Records excluded based on title not being relevant to topic of interest:

(n = )

**Screening**

Records excluded based on abstract not being relevant to development of the initial rough programme theory:

(n = )

Records excluded based on abstract not being relevant to development of the initial rough programme theory:

(n = )

Records excluded based on abstract not being relevant to development of the initial rough programme theory:

(n = )

Potential records relevant to the review screened for richness and rigour: full text screening

(n = )

Potential records relevant to the review screened for richness and rigour: full text screening

(n = )

Potential records relevant to the review screened for richness and rigour: full text screening

(n = )

Records excluded based on full-text having:

- little to no content that can add to theory development

(n= )

-data are less trustworthy and theory is less coherent.

(n = )

Records excluded based on full-text having:

- little to no content that can add to theory development

(n= )

-data are less trustworthy and theory is less coherent.

(n = )

Records excluded based on full-text having:

- little to no content that can add to theory development

(n= )

-data are less trustworthy and theory is less coherent.

(n = )

Records included for synthesis

(n= )

Records included for synthesis

(n= )

Records included for synthesis

(n= )

**Included**
