# Supplementary material for: Mental health policy implementation in low- and middle-income countries: a realist review protocol
Source: PLoS One. 2025 Mar 25;20(3):e0320420. doi: 10.1371/journal.pone.0320420 (PMC11936231; doi:10.1371/journal.pone.0320420)
Supplement: S4 File — (DOCX) [file pone.0320420.s004.docx]

**Supplementary File 4: Relevant and good enough screening procedure**

Screening Procedure

A. Import

1. Complete all searches and import the article references directly from the databases to Rayyan
2. The articles are saved as individual records and remove all duplicates.

B. Screening

1. Two reviewers will screen each record independently for 3Rs – relevance, richness and rigour.
2. Screen **abstracts and titles for relevance** to the review and rough initial programme theory (IPT) using the following criteria:
   1. CONTEXT (C)
      1. Methodological Studies

- Did the methodological paper describe national mental health policy or plans using theory, frameworks, models or implementation strategies? Yes/No (Y/N)
  - 1. Empirical studies
- Did the empirical study describe national mental health policy or plans and implementation strategies in low and middle-income countries (LMICs)? Y/N
- Did the empirical study employ any of the research designs: qualitative, quantitative or mixed methods? Y/N
  - 1. Grey literature
- Did the grey literature such as a report, policy, policy brief, or conference proceedings describe national mental health policy or plans and implementation strategies in (LMICs)? Y/N
  1. MECHANISMS (M)
     1. Did the study or grey literature give a description of perceptions, attitudes and behaviours of different stakeholders towards the resources for mental health policy implementation? Y/N
  2. OUTCOMES (O)
     1. Did the study or grey literature report on implementation outcomes? Y/N
     2. Did the study or grey literature report on mental health service provision outcomes? Y/N
     3. Did the study or grey literature report on mental health service users’ outcomes? Y/N

1. Finalise abstract and title screening outcomes using the following criteria:
   1. *Abstract screening*
      1. Is there a combination of two (CM or CO or MO) or more (CMO) eligibility criteria for context and/or mechanisms and/or outcomes in the abstract? Y/N
   2. *Title screening*
      1. Is there a combination of two (CM or CO or MO) or more (CMO) eligibility criteria for context and/or mechanisms and/or outcomes in the title?
2. For point 5, follow these additional steps:
   1. There are three final screening outcomes: include, exclude and maybe
      1. If the record has a combination of two or more eligibility criteria in the abstract/title, consider as relevant to the review. Assign the screening outcome as Include.
      2. If the record does not have a combination of two or more eligibility criteria in the abstract/title, consider as not relevant to the review. Assign the screening outcome as Exclude.
      3. If unsure of what outcome to assign, assign Maybe
3. Reconvene as reviewers and compare screening outcomes after the first 10%, 33%, 66% and 100% of title/abstract reviews are completed.
   1. For records with different screening outcomes, discuss and agree on the final screening outcome that has to be assigned.
   2. If an agreement is not reached, engage the study supervisor/an agreed upon third reviewer for their opinion. The final outcome to be assigned will be the majority count out of the three votes.
4. All screening outcomes assigned ”Include” for abstract/title screening will go through a round of full-text screening.
5. *Richness* of papers in contributing to IPT is described as follows^1^:

| Description | Criteria |
| --- | --- |
| Conceptually rich | studies with well-grounded and clearly described theories and concepts |
| Conceptually thick | studies with a rich description of a programme was provided, but without explicit reference to the theory underpinning it |
| Conceptually thin | studies with weak programme descriptions where discerning theory would have been problematic |

*Rigour* is the trustworthiness of the evidence source and the coherence of the programme theory and it can be conceptualised using either the TAPUPASM framework^[[1]](#footnote-1)^ or the following questions and answers^[[2]](#footnote-2)^:

TAPUPASM framework option

| Quality Criteria | Explanation |
| --- | --- |
| Transparency | Is the process of generating knowledge explicit and clear? |
| Accuracy | Are the claims made based on relevant information? |
| Purposivity | Do the methods achieve what they claim to achieve? Are they appropriate to achieve the aims and objectives? |
| Utility | Is there research appropriate to the decision-making setting? Does it provide answers to the practical questions? |
| Propriety | Is there research legal and ethical? |
| Accessibility | Does it meet the needs of those seeking the knowledge? |
| Specificity | Does the research generated consider and apply to source specific standards? |
| Modified Objectivity | Does the research review a range of evidence and draw the most likely conclusions based on this? |

Questions and answers option

- ***Q:*** *Is rigour assessed at the level of the evidence source or the programme theories developed?*

***A:****Rigour should be assessed at both the evidence source and programme theory levels.*

- ***Q:****What does ‘rigour’ look for?*

***A:****At the evidence source level, we are asking if the data are****trustworthy****. This may consider the methodological process and credibility of the source. At the theory level, we are asking if the theory is****coherent****. A coherent theory is consilient (explains the data), simple (makes few assumptions), and analogous to substantive theory (aligns with existing credible theories).*

- ***Q:*** *What is the impact or influence of doing a rigour assessment?*

***A:****The purpose of assessing rigour during a realist review is to ensure that the data and theory are rigorous so that the resulting recommendation(s) can inform evidence-based practice. When the rigour of the evidence source is low (i.e. the data are not trustworthy), reviewers can overcome this by triangulating the data with additional and/or more credible sources by revisiting previously excluded sources or conducting a new search for evidence. When the rigour at the theory level is low (i.e. the theory is not coherent), reviewers can search for additional evidence to further explain, refine, or refute this theory to make it more rigorous. If the rigour cannot be improved through additional data or if this is beyond the scope of the review, reviewers are urged to be transparent and present the theory as having ‘less’ rigour. This can be followed with suggestions for further primary research to redress the gap in available evidence. However, if a review is intended to inform policy/practice, caution should be given in reporting any recommendations based on non-rigorous theory.*

- ***Q:****What is the relationship between rigour at the evidence source level and the programme theory level?*

***A:****Rigorous data (trustworthy) does not necessarily equate to a rigorous theory(coherent). Less rigorous data or theory may call for additional evidence searches, as described in the previous question.*

- ***Q:****How do I be transparent with my methods for evidence appraisals, especially when considering a limited publication word count?*

***A:****Be explicit about the considerations given to both the data and theory-levels in appraisals and what was considered. Utilise boxes/tables/illustrations and supplementary materials to show how data was triangulated or supported where necessary.*

Screen **full text for richness and rigour** to the review and rough initial programme theory (IPT) using the following criteria

| Screening outcome | Rating | Criteria |
| --- | --- | --- |
| Include | Good | Papers in this category have a CMO component to them. Excerpts from the papers have sufficient content that can add to theory development. Data are trustworthy and theory is coherent. |
| Maybe | Fair | Papers in this category have a CM or CO or MO component to them. Excerpts from the papers have moderate content that can add to theory development. Data are trustworthy and theory is coherent. |
| Exclude | Poor | Papers in this category have a CM or CO or MO component to them. Although relevant in respect to MHP implementation, excerpts from the papers have little to no content that can add to theory development. Data are less trustworthy and theory is less coherent. |

1. Reconvene as reviewers and compare screening outcomes after the first 10%, 33%, 66% and 100% of full-text reviews are completed.
   1. For records with different screening outcomes, discuss and agree on the final screening outcome that has to be assigned.
   2. If an agreement is not reached, engage the study supervisor/an agreed upon third reviewer for their opinion. The final outcome to be assigned will be the majority count out of the three votes.
2. Papers with a final screening outcome of “Include” for richness and rigour will be include in the data synthesis.

1. Ryan, G. and Rutty, J., 2019. Philosophy and quality? TAPUPASM as an approach to rigour in critical realist research. *Nurse researcher*, *27*(1). [↑](#footnote-ref-1)
2. Dada, S., Dalkin, S., Gilmore, B., Hunter, R. and Mukumbang, F.C., 2023. Applying and reporting relevance, richness and rigour in realist evidence appraisals: advancing key concepts in realist reviews. *Research synthesis methods*, *14*(3), pp.504-514 [↑](#footnote-ref-2)
