# Supplementary material for: Mental health policy implementation in low- and middle-income countries: a realist review protocol
Source: PLoS One. 2025 Mar 25;20(3):e0320420. doi: 10.1371/journal.pone.0320420 (PMC11936231; doi:10.1371/journal.pone.0320420)
Supplement: S5 File — (DOCX) [file pone.0320420.s005.docx]

**Supplementary File 5: Data extraction form for background searches**

(version 0.1)

1. Document ID (chronologically i.e. 1,2, etc)
2. Authors

- Names, year published

1. Document type (methodology papers i.e. policy implementation theories OR empirical papers i.e. qualitative, quantitative or mixed methods studies OR grey literature i.e. reports, policies, briefings, conference proceedings)
2. Context (what and where),

- Describe briefly the main purpose of the document;
- Health system level(s) - national, provincial, district or not stated *(*only if document type is an empirical paper)*
- Policy level (s) - national, provincial, district or not stated *(*only if document type is a policy)*
  - Aspects measured in the policy
- Country – name of country or not stated
- Population – description of population or not stated
- Implementation strategy – description of implementation strategy or not stated *(*only if document type is an empirical paper or grey literature)*
- Intervention – description of intervention or not stated *(*only if document type is an empirical paper or grey literature)*
- Control– description of control or not stated*(*only if document type is an empirical paper or grey literature)*

1. Mechanisms (how and why)

- Page number from which mechanism was drawn
- First few words of sentence from which mechanism was drawn

1. Outcomes

- Implementation outcomes
- Health service provision outcomes
- Patient-level outcomes

1. Design/methods

- Design/methods used
- Overall quality of design/methods -high, medium, low

1. Tools

- Tool used
- Tool described - Yes/No
- Overall quality of tool - high, medium or low

1. Sample

- Sampling method
- Sample size

**Notes:*

1. Questions 1 –6 only apply to methodological papers
2. Questions 1 – 7 only apply to grey literature
3. Questions 1 – 9 apply empirical papers
